# Development of a Novel Thermoplastic Tubing, FP-FLEX™, and Single-Use Freezing Bag for Working Cell Banks Enabling Closed-System Processing to Temperatures as Low as -196°C

**DOI:** 10.1186/1753-6561-9-S9-P58

**Published:** 2015-12-14

**Authors:** Dominic Clarke, Doug Norris, Bradley Bennett

**Affiliations:** 1 Charter Medical, Ltd., Winston-Salem, NC, 27103, USA

## Introduction

Working cell banks (WCB's) are commonly applied to initiate cell culture manufacturing campaigns for production of recombinant or therapeutic proteins. These campaigns typically begin with inoculation of cells previously cryopreserved in cryovials or ampoules. The baseline process proceeds from thaw of cell bank cryovials to scaling up through the seed train and inoculum train, followed by fed-batch production. While cryovials are typically used in the development of WCB's and initiation of manufacturing campaigns, they are not optimal for the growing demands of commercial production.

Cryovials are small on average (1-2mL/vial) and filling/removal is performed through an open screw cap. This process results in manual operations and the use of many culture vessels, resulting in the risk of contamination and potential campaign-to-campaign variability. Single-use (disposable) bags have been investigated more recently as a possible solution to minimize open handling steps and to shorten seed train scale-up. Bags offer larger storage volumes, but also come assembled with thermoplastic tubing for sterile connections. Wide-spread adoption of single-use bags for WCB applications has not been observed to this point as currently available tubing and connections can't hold up to the demands (break or can't be welded) when stored/transported at frozen or cryogenic (-196°C) temperatures.

To overcome these challenges, novel thermoplastic tubing was developed to balance both the flexibility and robustness demands of cryogenic storage and tube welding characteristics necessary for sterile closed-system processing. The new FP-FLEX™ tubing can be frozen and maintained at cryogenic temperatures, thawed and sterile welded to other thermoplastic tubing (C-Flex®).

## Methods and Results

A selection of different thermoplastic elastomer combinations were investigated in an effort to discover the optimal blend of materials to support the flexibility and robustness requirements during frozen storage and sterile welding needs for processing. A novel manufacturing method was also designed to enable welding of the FP-FLEX™ tubing directly to the single-use bags. This was critical to support a completely unitized, closed design. Studies were then carried out to determine both durability and functional utility of the FP-FLEX™ tubing for frozen storage and processing applications.

Testing was carried out using 500mL nominal fill bags having (1/4" OD × 1/8" ID) FP-FLEX™ lines (8" L) and compared to bags having PVC tubing (standard application). For LN2 studies, all bags were filled to 140mL (+/- 5mL) with water and placed into aluminum cassettes prior to storage with tubing attached. Simulated drop tests were performed to test overall durability. Each Bag was pulled from the LN2 tank and immediately dropped horizontally 4X from a distance of 1ft, thawed and evaluated for damage. 10 of 10 bags with FP-FLEX™ tubing were completely intact while 10 out of 10 bags with PVC tubing were broken. A single drop (FP-FLEX™ bags only) from 2ft and 3ft resulted in a similar outcome (Table 1). Furthermore, simulated frozen transportation studies were performed (in LN2) to ensure products could be shipped and maintain integrity. All samples passed without damage (data not shown).

**Table 1:** FP-FLEX Integrity Testing.

Handling/Drop Test		
**Product**	**Drop Height**	**Result**
PVC Tubing	1 foot	0/10 pass
FP-FLEX™	1 foot	10/10 pass
FP-FLEX™	2 feet	5/5 pass
FP-FLEX™	3 feet	5/5 pass
**Post Freeze/Thaw Welding FP-FLEX™ to C-Flex**® **Testing**		
**Property**	**Test Protocol**	**Result**
Integrity	Pressure leak test (1psi)	21 samples/pass
Flow Rate	Welded junction flow rate ≥500mL/minute	21 samples/pass
Weld Strength	Freeze/thaw FP-FLEX™ welded to C-Flex®	Ave = 12.85 lbf

FP-FLEX™ and Freeze-Pak™ are trademarks of Charter Medical, Ltd. Cflex® is a registered trademark of Saint-Gobain Performance Plastics Corporation.

Finally, post freeze/thaw weld strength and integrity testing was performed to evaluate sterile welding capabilities of FP-FLEX™ tubing post-thaw (Table 1). Tubing was capable of welding directly to C-Flex® following freezing using standard sterile welders with an average weld strength of 12.85 lbf. Flow rates of 0.5L/min were tested and achieved successfully.

## Conclusions

**Figure 1:**
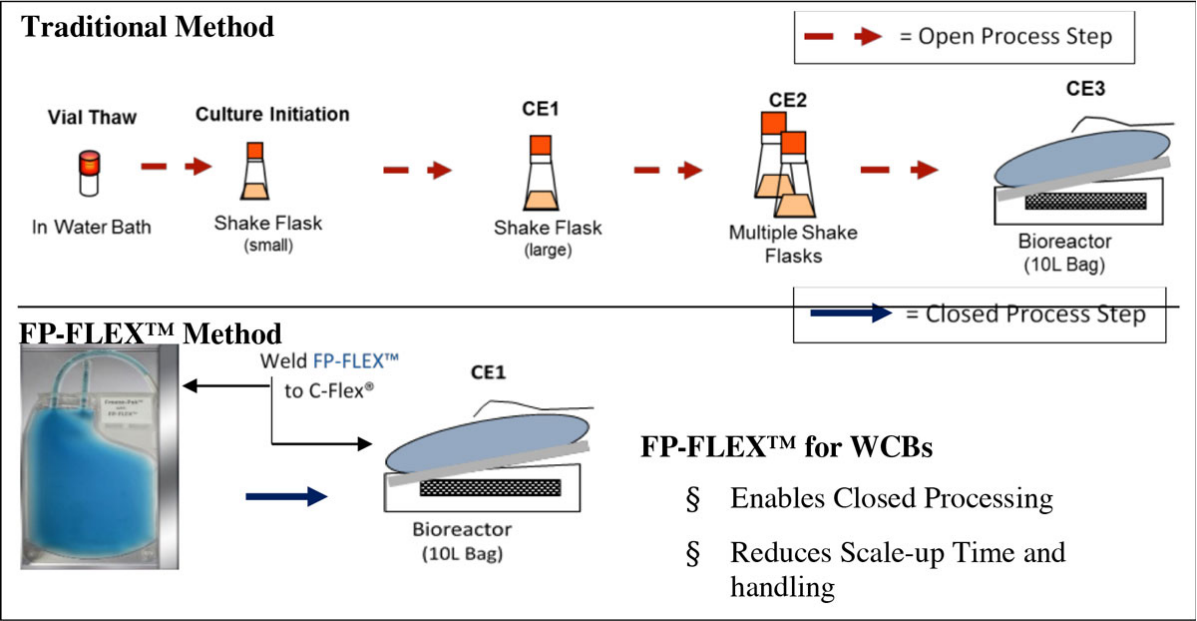
**Traditional method compared to new FP-FLEX™ method for WCB's**.

WCB's are commonly used for seed train manufacturing of therapeutic products. Traditional vials represent an open manufacturing process and are also limited to small volumes which contribute to lengthened production campaigns. The new FP-FLEX™ tubing has been designed and shown herein to meet the critical processing requirements for WCB's (Figure 1).

Freezing/Storage/transport to as low as -196°C

Weldable to C-Flex® post freeze/thaw

Compatible with standard tube welding and sealing devices

Closed-system aseptic transfer via tube-to-tube connection

The FP-FLEX™ tubing and Freeze-Pak™ bag represent a closed-system solution enabling frozen storage, sterile connection and reduced scale-up time for therapeutic production.

